# Mechanisms of stomatal development: an evolutionary view

**DOI:** 10.1186/2041-9139-3-11

**Published:** 2012-07-06

**Authors:** Anne Vatén, Dominique C Bergmann

**Affiliations:** 1 Department of Biology, Stanford University, Stanford, CA, 94305-5020, USA; 2 Institute of Biotechnology/Department of Bio and Environmental Sciences, University of Helsinki, Helsinki, FIN-00014, Finland; 3 Howard Hughes Medical Institute, Stanford, USA

**Keywords:** Stomata, Plant evolution, bHLH transcription factors, Arabidopsis, Maize, Physcomitrella, Rice, Ligand receptor signaling, Cell polarity, Asymmetric cell division

## Abstract

Plant development has a significant postembryonic phase that is guided heavily by interactions between the plant and the outside environment. This interplay is particularly evident in the development, pattern and function of stomata, epidermal pores on the aerial surfaces of land plants. Stomata have been found in fossils dating from more than 400 million years ago. Strikingly, the morphology of the individual stomatal complex is largely unchanged, but the sizes, numbers and arrangements of stomata and their surrounding cells have diversified tremendously. In many plants, stomata arise from specialized and transient stem-cell like compartments on the leaf. Studies in the flowering plant *Arabidopsis thaliana* have established a basic molecular framework for the acquisition of cell fate and generation of cell polarity in these compartments, as well as describing some of the key signals and receptors required to produce stomata in organized patterns and in environmentally optimized numbers. Here we present parallel analyses of stomatal developmental pathways at morphological and molecular levels and describe the innovations made by particular clades of plants.

## Review

### Introduction to stomata and stomatal pattern

Plants conquered land more than 400 million years ago. In the fossil record, the appearance of these pioneer species is contemporaneous with the appearance of structures on their surfaces called stomata. Each stoma (plural, stomata) consists of paired epidermal guard cells, a pore between them and an airspace in the photosynthetic mesophyll tissue subtending it. The function of stomata is to regulate gas exchange between the plant and its surroundings. On short timescales (minutes to hours), the opening and closing of the stomatal pore by turgor-driven changes in guard cell shape is a key regulatory step in maintaining water and carbon dioxide balance. Work from many laboratories has defined the intracellular signal transduction cascades that mediate changes in pore size in response to hormone and environmental signals [1].

The current view is that stomata arose only once during evolution [2]. In early land plants, stomatal density was low [3]. During intervening millennia, the stomatal density (SD, number of stomata/unit leaf area) increased, probably in response to reduced aerial CO_2_ concentration [4]. The stomatal complex has been fine-tuned by several innovations including recruitment of neighboring subsidiary cells to facilitate stomatal opening/closing, relocation of stomatal complexes under protective epidermal cells and incorporation of multiple asymmetric cell divisions in precursors to create a variety of stomatal distributions. Despite the variation, the basic core structure has remained unchanged: two guard cells flank the stomal pore. In nearly all species, two stomata are separated at least by one non-stomatal cell, an arrangement thought to be essential for efficient opening and closing. Stomata are located on aerial organs including leaves, stems, flowers, fruits and seeds and they develop gradually during organ growth such that young organs have fewer total stomata than mature organs, though SD often decreases as the neighboring epidermal cells expand during maturation. The frequency and positioning of stomata are organ and species-specific characters, but are also affected by environmental factors.

### Innovations

**Figure 1:**
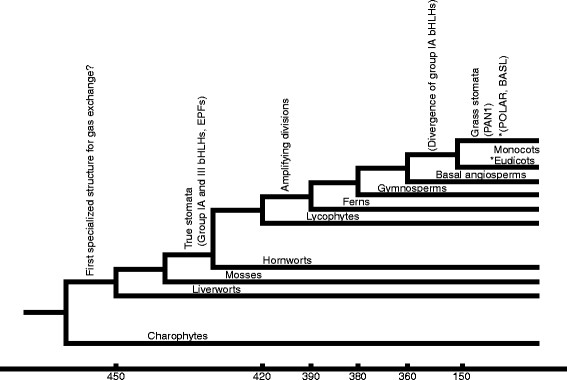
**Divergence of major land plant lineages and appearance of stomatal characteristics.** Phylogenetic tree of extant land plants indicating positions of major innovations in the evolution of stomata, following Ruszala *et al.*[5]. Those in brackets indicate predicted appearance of stomatal development regulatory genes. Numbers on the x-axis refer to multiples of millions of years

**Figure 2:**
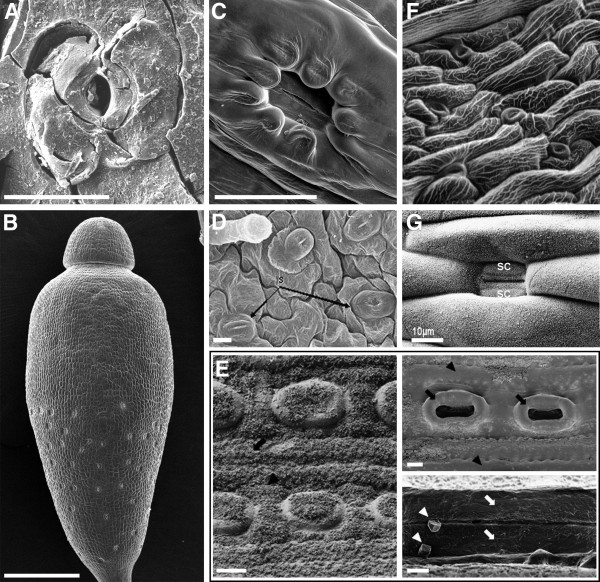
**Representative stomatal complexes and patterns from different species.** (**A**) Scanning electron micrograph (SEM) of Silurian fossil stoma displaying common morphology. Scale bar, 20 μm [3]. (**B**) SEM of moss *Bryum capillare* sporangium with stomata visible on the lower half. Scale bar, 600 μm [2]. (**C**) SEM of moss *Bryum capillare* sporangium stoma sunken below epidermal cells. Scale bar, 50 μm [2]. (**D**) SEM of fern *Thelypteris ovata* var. *lindheimeri* (sporophyte) leaf with stomata separated by pavement cells. Scale bar, 10 μm; s, stomata [6]. (**E**) Left panel, field emission SEM of *Pinus koraiensis* (gymnosperm) stomata arranged in rows on needle surface; granular material is surface wax. Scale bar, 10 μm. Upper right, dewaxed stomata. Scale bar, 10 μm. Lower right, dewaxed guard cells (arrows) within an epistomatal chamber. Scale bar, 2 μm [7]. (**F**) SEM of dicot *Arabidopsis thaliana* stomatal pattern in the sepal. (**G**) SEM of monocot *Poa annua* stoma, with subsidiary cells (sc) flanking the narrow guard cells. Scale bar, 10 μm [8]

Paleobotanical analyses utilizing the fossils of the early land plants and their currently living descendants have been combined with phylogenetic analyses to address the origins of stomata (Figures 1 and 2A). Liverworts, mosses and hornworts comprise the bryophytes, a basal land plant group. Liverworts do not have stomata; gas exchange is facilitated by epidermal air pores, structures whose development and morphology differ from stomata. Stomata are found in mosses and hornworts, making it likely that liverworts diverged from other bryophytes before the origin of stomata. Intriguingly, in extant bryophytes, both guard cell morphology and regulation of pore aperture can closely resemble higher plant stomata.

**Figure 3:**
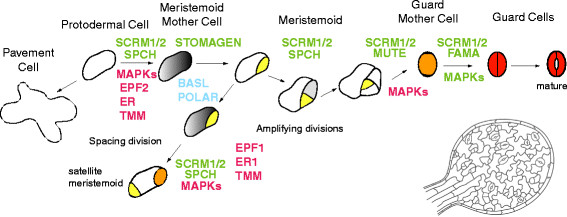
**Stomatal development in *Arabidopsis ***. Diagram of major stages in stomatal development with place of action of the subset of regulatory genes discussed in this review noted. Positive regulators are written in green, negative regulators in red and polarity regulators in blue. Not all genes known to regulate stomata are presented. The image of the young leaf in the lower right corner is to represent the dispersed nature of stomatal lineage initiation. Color code: yellow, meristemoid; orange, guard mother cell; red, guard cell; grey, meristemoid mother cell (MMC)

In the most recently derived plant group (angiosperms, or flowering plants) there is a dedicated epidermal lineage that produces stomata. In dicot plants, such as the research model *Arabidopsis*[9], these lineages are initiated from various sites on the leaf (Figure 3). In each lineage, a committed protodermal cell called the meristemoid mother cell (MMC) divides asymmetrically to give rise to a larger stomatal lineage ground cell (SLGC) and smaller meristemoid. The meristemoid undergoes one to three asymmetric divisions (amplifying divisions) before it differentiates into a guard mother cell (GMC). Later, SLGCs can also divide asymmetrically and produce more meristemoids (spacing divisions). The GMC divides symmetrically to create two guard cells, and in some species the GMCs recruit neighboring subsidiary cells. These subsidiary cells can provide mechanical assistance and a source of ions required for guard cell movement. Amplifying and spacing divisions and subsidiary recruitment all require cell to cell communication and together they contribute to pattern. The frequency with which cells participate in these division types can be modified to yield the extraordinary diversity of stomatal patterns seen in nature [10] (Figure 2B2D and 2F).

**Figure 4:**
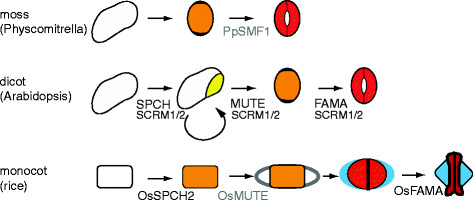
**Comparison of the molecular and morphological features of stomatal development in *Arabidopsis *and representatives of the grasses and mosses for which molecular data exist.** Presentation of a simplified stomatal lineage displaying only cell identities (in the same color codes as Figure 3), with the addition of blue to mark the subsidiary cells in monocots. Genetic regulators of the processes are included at their points of action, with black text indicating that there is direct functional evidence supporting the placement and grey text representing inferences from cross-species complementation tests. The curved arrow in the dicot lineage represents the continued asymmetric amplifying divisions made by meristemoids

Monocots exhibit a strong base to tip gradient of leaf differentiation with stomata-producing cell lineages forming at the base of the leaf. Asymmetric cell divisions produce GMCs without prior transit through a self-renewing meristemoid stage (Figure 4). Protodermal cells in files flanking the GMC polarize towards the GMC and divide asymmetrically giving rise to subsidiary cells. After this, the GMC divides to produce guard cells that exhibit a novel flattened or dumbbell-shaped morphology (Figure 2G). In monocots like *Tradescantia,* overall stomatal pattern can be refined when GMCs change fate and differentiate into epidermal cells [11]. This fate change is dependent on distance from neighboring stomata, suggesting an inhibitory communication mechanism.

Given the current patterns and developmental processes associated with stomata of flowering plants, what were their origins? In the simplified ontogeny seen in some mosses, stomatal development involves a single asymmetric cell division giving rise directly to a GMC (Figure 4). This GMC may not divide completely, as seen in *Funaria hygrometrica* where two guard cell nuclei are separated by an incomplete cell wall [12], or may break the spacing rule as in *Polytrichastrum formosum* where stomata sometimes form next to each other. Already in mosses, diverse stomatal morphologies are seen [2]. Several (both early- and late-divergent) moss species also lack stomata [13]. The function of stomata in these plants may also be unusual, for example the basal genus *Sphagnum* displays pseudostomata which might function in spore desiccation rather than typical CO_2_ acquisition [14].

The appearance of amplifying divisions in ferns provided novel mechanisms to control cell number as well stomatal density and to produce specialized subsidiary cells [15,16]. Here an epidermal cell may go through one or two asymmetric cell divisions before it differentiates into a GMC. Subsidiary cells in gymnosperms (for example, pines) can arise from meristemoid divisions or division of protodermal cells next to stomata, or both. In *Pinus strobus* and in *Pinus banksiana*, meristemoids divide once symmetrically to generate a GMC and a subsidiary cell [17]. The subsidiary cell, as well as neighboring epidermal cells, expands in a polar fashion over the GMC. As a result the GMC, and later the guard cell pair, is overlaid by a group of epidermal subsidiary cells of mixed origin and in addition, is closely connected to hypodermal subsidiary cells. Also in gymnosperms, we begin to see stomata incorporating some of the biochemical innovations of this group (Figure 2E). For example, subsidiary cells display thick, waterproof cuticles, and the guard cells become reinforced with lignin, a cell wall polymer that is not present in bryophytes [18].

Despite the benefits of stomata-mediated gas-exchange, some plant lineages have lost stomata. This is sometimes facultative; for example among heterophyllic species, two alternate leaf forms are made, depending on whether the leaf is submerged in water or airborne. In these species, leaf submergence leads to elimination of stomata [19]. Some parasitic plants, whose sources of fixed carbon are their hosts, may also lose or inactivate their stomata [16]. Other plant groups, like the small, predominantly aquatic isoetes, have members that have lost stomata completely. The astomatous isoetes gain CO_2_ from the sediment via their extensive root system [20]. Isoetes perform a variant of photosynthesis common among cacti (crassulacean acid metabolism (CAM)), in which separation of particular biochemical reactions allow (stomatous) plants to only open stomata during the night to decrease water loss. Use of a root-derived carbon source enabled astomatous isoetes to fix carbon continuously without a threat of stomata-related water loss. In general, astomatous species are small and only exist in a narrow growth environment. It has been suggested that functional stomata allow plants to develop to larger sizes and to adapt to a wider range of growth conditions [21].

### Pathways for stomatal development in *Arabidopsis*

The regulation of stomatal development is best understood at a molecular level in *Arabidopsis.* Here, individual cell fate transitions in the stomatal lineage are promoted by three closely related basic helix-loop-helix (bHLH) transcription factors, SPEECHLESS (SPCH), MUTE and FAMA [22-24] (Figure 3). These bHLHs are expressed in the stomatal lineage, each in a specific developmental window, and each of them is absolutely required for stomata formation. SPCH is expressed in subset of young epidermal cells, often in two adjacent cells [24] and SPCH expression is dynamic. After an asymmetric cell division, SPCH disappears from the SLGC, but remains in the meristemoid, which continues asymmetric cell divisions [25]. Loss of *SPCH* leads to a complete loss of the stomatal lineage whereas overexpression of *SPCH* leads to ectopic asymmetric cell divisions [23,24,26]; thus it is required for entry into the stomatal lineage. MUTE is expressed in late meristemoids and is required for exit from the amplifying division stage, and it promotes the meristemoid to GMC transition [23,24,26]. FAMA is expressed in the GMC and in immature guard cells. Overexpression of FAMA leads to ectopic formation of unpaired guard cells indicating that FAMA promotes stomatal cell fate while restricting (symmetric) divisions [22].

Proteins encoded by the paralogous bHLHs, *INDUCER OF CBF EXPRESSION1/SCREAM (ICE1/SCRM)* and *SCRM2*, form heterodimers with SPCH, MUTE and FAMA and promote all three stomatal fate transitions [27]. A semidominant *scrm-D* mutant converts the epidermis into stomata, a phenotype identical to MUTE overexpression, whereas double mutants of *ICE1/SCRM* and *SCRM2* resemble *spch*[27]*.* Interestingly, ICE1/SCRM has been shown to be involved in cold stress response [28]. Since stomatal development is regulated by both environmental [21] and developmental factors [29], it is possible that ICE1/SCRM is a cross-regulatory node where several signaling pathways are integrated to direct stomatal development.

More signal integration occurs via mitogen-activated protein kinases (MAPKs) which regulate stomatal development and stress responses through a three-step phosphorylation cascade. MAPK kinase kinase YODA, MAPK kinases (MKK4/5/7/9) and MAPKs (MPK3/6) are essential for normal stomatal spacing [30-32]. SPCH is a direct target of MAPK-mediated phosphorylation and this serves to negatively regulate SPCH activity [33]. The MAPK pathway also regulates the later stages of stomatal development, but the targets have not been identified. More complexity arises from the recent finding that signaling intermediates from the steroid hormone brassinosteroid (BR) pathway phosphorylate both YODA [34] and SPCH [35]. Interestingly, BR-modulated phosphorylation as mediated through YODA and SPCH actually produces opposite stomatal phenotypes. Combined with other evidence that SPCH is differentially methylated under certain environmental conditions [36], we are seeing just hints of the complex interactions and precise tuning to which the early parts of the stomatal pathway may be subjected.

Upstream of the intracellular signalling cascades, genetic studies have revealed that stomatal spacing is regulated by secreted peptides of the EPIDERMAL PATTERNING FACTOR-LIKE (EPFL) family [37-41], by three leucine-rich repeat receptor kinases (LRR-RLKs), ERECTA (ER), ERECTA-LIKE 1 (ERL1) and ERL2 [42] and one LRR-receptor-like protein, TOO MANY MOUTHS (TMM) [43,44]. Members of the ER-family (ERf) are broadly expressed and their absence leads to severe stomatal overproliferation and mispatterning, as well as pleiotropic growth phenotypes, indicating that they regulate multiple developmental processes [42,45]. ER acts predominantly as a negative regulator of entry divisions whereas ERL1 and ERL2 control later stages [42]. TMM is expressed in the early stomatal lineage and, thus far, only roles in stomatal development have been described [44].

EPF1 and EPF2 peptides are stomatal lineage-expressed and regulate the number and orientation of asymmetric divisions [37-41]. Loss of either *EPF1* or *EPF2* results in more stomata, but mutant and overexpression phenotypes indicate that EPF2 prevents entry into the stomatal lineage whereas EPF1 acts later. Their paralogue, *STOMAGEN/EPFL9*, by contrast, is expressed in the underlying cell layer (mesophyll) and travels to the epidermis to promote stomatal differentiation [46,47]. EPF1, EPF2 and STOMAGEN require the receptor TMM for full activity [37-41,46,47]. Surprisingly, the function of three other EPFLs, EPFL6/CHALLAH (CHAL), EPFL4 and EPFL5, is inhibited by the presence of TMM [40]. Although CHAL was originally identified by its stomatal phenotype in a *tmm* background [41], *CHAL/EPFL4/EPFL5* are expressed in internal tissues and their loss leads to a compromised growth phenotype resembling loss of *ER*[40]. Thus, they likely represent ligands for ER’s non-stomatal roles. The identification of EPF family members with distinct developmental roles has led to interesting models of how signaling specificity is achieved by using the non-kinase receptor TMM to modulate ligand interactions with the ERf kinases in specific tissues [40]. Recently, elegant biosensor approaches demonstrated ER and ERL1 primarily bind EPF2 and EPF1, respectively, *in vitro*[48] and that *in planta*, TMM can heterodimerize with ER and ERL1. Clarifying the physical interactions and *in vivo* activities of the four receptors with the 11 members of the EPF family looks to be an exciting future area of research in *Arabidopsis*. Homologues of ERf, TMM and EPFLs are found in diverse species, including monocots and mosses, indicating that the potential for conserved signaling systems exist. To date, however, no experimental information is available outside of *Arabidopsis*.

Stomatal lineages in *Arabidopsis* are established by asymmetric cell divisions, and these unusual and unequal divisions involve several other novel, plant-specific, proteins: BREAKING OF ASYMMETRY IN THE STOMATAL LINEAGE (BASL) [49] and POLAR LOCALIZATION DURING ASYMMETRIC DIVISION AND REDISTRIBUTION (POLAR) [50]. BASL displays dynamic spatiotemporal localization in the stomatal lineage. Before asymmetric cell division, BASL is detected in both the nucleus and at the cell periphery distal to the cell division plane. After the division, daughter cells inherit BASL in a manner that defines their fate: nuclear localization (differentiation to guard cells), peripheral localization (differentiation to pavement cell), or both (continued asymmetric cell divisions) [49]. *BASL* mutants display misoriented asymmetric cell division and overexpression of BASL leads to ectopic outgrowths in the positions where BASL is peripherally concentrated [49]. Hence, it seems possible that BASL controls or mediates cell polarity during asymmetric cell division in the stomatal lineage. POLAR shares some features of the BASL localization pattern; it is peripheral and distal to the cell division site before asymmetric cell division and shows unequal behaviors in the daughters, disappearing from the larger daughter and being upregulated in the smaller, meristematic, daughter [50]. Although no phenotypes have been ascribed to loss of *POLAR*, its localization is dependent on *BASL* suggesting that they act in the same pathway [50].

### Additional rules for monocot stomata

One of the major differences between dicot and monocot (specifically, grass) stomatal pathways is that, in the latter, subsidiary cells are recruited from cell files flanking the stomatal lineage. This process requires the generation of a highly polarized cell division that is specifically oriented toward the GMC. After formation of a GMC (itself formed by asymmetric division within the stomatal lineage), neighboring subsidiary mother cells (SMCs) divide asymmetrically to produce small subsidiary cells next to the GMC (Figures 2G and 4). SMC polarization involves localization of F-actin patches along the cell wall flanking the GMC, and nuclear migration towards the actin patches [51]. In maize, actin patches co-localize with an LRR-RLK protein, PANGLOSS 1 (PAN1) [52]. Despite shared roles in stomatal development, PAN1 is not in the same LRR-kinase family as ERf and there are no polarly localized LRR-kinases implicated yet in *Arabidopsis* stomatal development. Loss of *PAN1* leads to mislocalization of actin and the nucleus. This disrupts asymmetric cell divisions and results in abnormal subsidiary cells [52,53]. Recently, the actin regulators Rho of plants 2 (ROP2) and ROP9 were shown to localize polarly in SMCs and to promote SMC polarization [54]. PAN1, ROP2 and ROP9 interact and localization of ROP2 and ROP9 is dependent on PAN1, but PAN1 localization is independent of ROPs. It is attractive to speculate that these proteins work in a common pathway to receive polarity cues and translate them into the cellular reorganization necessary for SMC polarization [54].

### Evolution of stomatal regulators

#### *Arabidopsis* stomatal bHLH genes are in stomatal-producing plant lineages

As described above, in *Arabidopsis*, five bHLH genes are major determinants of the identities and behaviors of different stomatal lineage precursors. SPCH, MUTE and FAMA are fairly restricted in their expression pattern to subsets of the stomatal lineage whereas ICE1/SCRM and SCRM2 are expressed throughout the lineage and in additional non-stomatal lineage cells. When considering the diversity of stomatal pattern in nature, it is interesting to think about how the expression, regulation and function (and existence) of this class of regulators may change. Moreover, one might ask whether the heterodimeric partnership between SPCH, MUTE and FAMA with ICE1/SCRM and SCRM2 could be ancient or whether this is a new innovation.

The bHLH family is characterized by a conserved DNA binding region, but there are easily recognizable sub-families within*. SPCHMUTE* and *FAMA* belong to the group IA bHLHs [55] whose genic intron/exon structure and protein C-termini are distinctive enough to serve as high confidence group characters throughout the flowering plants and out to *Selaginella* (a model lycophyte) and *Physcomitrella* (a model moss) [56]. Distinction among individual 1A members is only clear within the flowering plants. ICE1/SCRM and SCRM2 are members of group III and representatives of this group are found in many clades back to the mosses (http://www.phytozome.net/). In the incomplete transcriptome and genome sequences from plant lineages predating the emergence of stomata, neither group IA nor group III bHLH genes are obvious [56] (C MacAlister, *personal communication*). Based on sequences currently available, in all cases where group 1A members can be distinguished, there is also a group III bHLH, suggesting that their partnership can be ancient. A group 1A homologue from *Physcomitrella* can partially complement *Arabidopsis mute* and *fama*, but not *spch* mutants [56]. These cross species complementation results are interesting in light of the shortened pathway for development of stomata in *Physcomitrella;* in this moss, no early asymmetric divisions are evident and instead a single GMC is specified and undergoes incomplete cytokinesis to form two connected guard cells (Figure 4). This pathway would require MUTE-and FAMA-like fate promoting activities, but not the division-promoting activity of SPCH [56].

In grasses, the positions of stomata are determined and fixed at early stages of leaf development and amplifying divisions are not present. In fact, only the differentiation (GMC to guard cells) step is similar between grasses and *Arabidopsis* (Figure 4). Nonetheless, *SPCHMUTE* and *FAMA* genes can be identified in the genomes of maize, rice and *Brachypodium.* There has also been a duplication of *SPCH* in these plants [57]. Perhaps due to the different stomatal ontogenies, however, the rice homologues OsSPCH1/2 are expressed very early during plant development, possibly before the production of stomatal lineage [57]. *OsSPCH2* mutants in rice, do, however, have reduced stomatal numbers and resemble weak mutant alleles of *AtSPCH*[57]. Overexpression of *OsMUTE* and *OsFAMA* recapitulates overexpression phenotypes of the *Arabidopsis* genes, indicating that their GMC and guard cell identity-promoting functions are conserved. Of the three genes, the only one acting at a stage common to stomatal development in both plant groups (the GMC to stomatal guard cell transition), *FAMA*, is most highly conserved in terms of expression pattern and loss of function phenotypes in both rice and *Arabidopsis*[57].

### New appearance of polarity regulators

Homologues of the cell fate regulators and many of the signaling components discussed above appear in many plant species [10,58,59]. In contrast, the two proteins shown to exhibit polarized localization in stomatal lineage cells of *Arabidopsis*, POLAR and BASL, do not. BASL does not resemble any other *Arabidopsis* proteins and only in the congeneric *A. lyrata* is there a significantly similar sequence. In *ArabidopsisPOLAR* is moderately similar to another gene (*POLAR-LIKE1*) and homologues of *POLAR* and *POLAR-LIKE1* can be found in closely related (dicot) species such as poplar (POPTR B9IL54). Already in rice, however, the sequence similarity becomes restricted to a very small domain of the proteins. It is interesting to consider whether this inability to find such homologues is because the function of BASL and POLAR is required only in the dicots, or because similar functions are carried out by different genes and the apparent uniqueness of these proteins represents either fast substitution rates or that their roles can be served by other proteins. For example, scaffold proteins that bind others together into complexes play important roles in polarity generation in yeast and animals, yet these scaffolds are often not well conserved at the sequence level and consist primarily of multiple interaction surfaces.

### Evolution of regulated stomatal pore opening

A developmental approach concerns itself with the correct specification and pattern of stomata. From a physiological point of view, however, the behavior of these final products is key. Modulation of the stomatal pore aperture depends on coordinated morphologies of the guard cell pair and, particularly in the case of the grasses, on the coordination of guard cells and the specialized subsidiary cells that are obligate parts of the stomatal complex [60]. For stomatal pore aperture to be optimized for daily and seasonal fluctuations in light, temperature, humidity and CO_2_ availability, the guard cells must be able to sense such environmental factors. Guard cells in angiosperms appear to sense many of these factors autonomously, and key kinases (OST1), phosphatases (PP2C) and receptors for the “drought stress” hormone, ABA (PYR1) have been identified in *Arabidopsis*[1]. Recent studies of CO_2_ and ABA responsiveness in non-vascular plants have come to different conclusions about when the sensing of these different environmental cues arose. Monitoring stomatal pore closure in response to ABA, [61] concluded that responsiveness to this hormone was a new feature and was absent in fern and lycophyte species. Other studies, however, provide evidence that ABA sensing may have arisen quite early. By cross-species complementation, Ruszala [5] and Chater [62] showed that the OST1 homologues from *Selaginella moellendorffii* and *Physcomitrella patens* could partially restore the ability of *Arabidopsis ost1* stomata to respond to ABA. Moreover, knockout of *PpOST1-1* significantly attenuated ABA response in *P. patens* stomata [62]. The differing conclusions from these studies could be due to the different “representative” species chosen, a general caution in evolutionary studies of this system that is also echoed in the behavior of maize and rice bHLHs [57].

## Conclusions

Stomatal development in *Arabidopsis* has been used as a model genetic system for the analysis of cell fate, cell polarity and cell to cell communication. The nature of the gene products identified in such analysis, coupled with the long tradition of evaluating the numbers and patterns of stomata in diverse plants for taxonomic purposes makes this system a useful natural laboratory to look at the parallel evolution of genes and developmental trajectories. As the number of completed plant genomes increases and tools for experimental manipulation of non-model species develop, we believe there will be an excellent opportunity to test the roles of candidate cell fate- and cell signaling factor-encoding genes in creating developmental diversity.

## Competing interests

The authors declare that they have no competing interests.

## Authors’ contributions

AV and DCB and designed the study, wrote the manuscript and prepared the figures. All authors read and approved the final manuscript.
