# Correction: Mechanisms of stomatal development: an evolutionary view

**DOI:** 10.1186/2041-9139-4-11

**Published:** 2013-04-04

**Authors:** Anne Vatén, Dominique C Bergmann

**Affiliations:** 1 Department of Biology, Stanford University, Stanford, CA, 94305-5020, USA; 2 Department of Biotechnology/Department for Bio and Environmental Sciences, University of Helsinki, Helsinki, FIN-00014, Finland; 3 Howard Hughes Medical Institute, Stanford, USA

## 

The following corrections correspond to our recently published review article [1].

Figure 1, the note of true stomata should be placed to the left of the branchpoint for mosses and hornworts, as both groups possess stomata.

Figure 2, Fig 2C is *Orthotrichum anomalum.* Full citations for images used in this figure are:

(A) Edwards D, Kerp H, Hass H: **Stomata in early land plants: an anatomical and ecophysiological approach.***J Exp Bot* 1998, **49**(Suppl 1)**:**255**–**278, reprinted by permission of Oxford University Press.

(B-C) Ligrone R, Duckett JG, Renzaglia KS: **Major transitions in the evolution of early land plants: a bryological perspective.***Ann Bot* 2012, **109:**851**–**871, reprinted by permission of Oxford University Press.

(D) de León MEM, Pérez-García B, Márquez-Guzmán J, Mendoza-Ruiz A: **Developmental gametophyte morphology of seven species of Thelypteris subg. Cyclosorus (Thelypteridaceae).***Micron* 2008, **39:**1351**–**1362, reprinted by permission of Elsevier.

(E) Kim KW, Lee S, Bae S, Kim P: **3d surface profiling and high resolution imaging for refining the florin rings and epicuticular wax crystals of Pinus koraiensis needles.***Microsc Res Tech* 2011, **74:**1166**–**1173, reproduced by permission of John Wiley and Sons.

(G) Koch K, Bhushan B, Barthlott W: **Multifunctional surface structures of plants: an inspiration for biomimetics.***Progress in Materials Science* 2009, **54:**137**–**178, reprinted by permission of Elsevier.
